# Evaluation of multidisciplinary simulation-based team training: the way forward for training ICU teams

**DOI:** 10.1186/2197-425X-3-S1-A860

**Published:** 2015-10-01

**Authors:** U Pietsch, H Schneider, W Schuhwerk

**Affiliations:** Kantonsspital St Gallen, Institute of Anaesthesiology & Intensive Care Medicine, St Gallen, Switzerland; Rea 2000, St Gallen, Switzerland

## Introduction

In May 2010, The Helsinki Declaration for Patient Safety in Anaesthesiology was launched by the two major anaesthetic societies in Europe. The ICU environment is especially unforgiving for mistakes due to the multidisciplinary, time-critical nature of care and vulnerability of the patients. Human factors account for the majority of adverse events and a sound safety climate is therefore essential [1].

Endangerment of patients by deficits in the field of Human Factors (HF) is especially tragic, as it should be avoidable in most cases. Simulation trainigs provide a valuable tool to train the management of complex medical situations thus reducing the occurrence of fatal errors and increasing patient safety.

We demonstrate the effects of trainigs by the means of a “ Pre- and Post-Training Self-Evaluation Questionnaire“ [2]. In addition, the debriefing after each training was evaluated by a standardised questionnaire (Debriefing Assessment for Simulation in Healthcare, DASH©).

## Objectives

Over the period of one year, nearly every health professional of our SICU (120 nurses and 15 physicians) underwent a simulation training consisting of 5 hours of training for each 8 participants ending with a video-assisted debriefing. The focus of the training was on the area of CRM and HF. In every training, typical medical emergencies such as anaphylaxia, critical increase of icp, severe hypotension, tension pneumothorax after the insertion of a central venous catheter, were reproduced.

## Results

So far, the first 75 questionnaires (65 nurses, 10 physicians ) have been evaluated regarding pre- and post training self evaluation. On a scale ranging from 1 (best) to 6 (worst) the overall rating of the training was 1.04. The multidisciplinary approach was rated 1.22. We could demonstrate that the participants felt they could improve their skills in all of the items that were assessed. The multidisciplinary approach of the training was esteemed by the majority of the participants. The debriefing was rated with 6.59 (scale ranging from 1, bad to 7, very good) in the DASH©-assessment.

## Conclusions

Multidisciplinary simulation-based educational trainig is feasible and improves self-estimated competence and awareness of CRM and HF in a medical complex ICU setting . This could have the potential to impact patient outcome. Interprofessional simulation trainings lead to a subjective increase of self-assuredness in the management of complex situations on ICU. A training within the unit is regarded mainly positively by the participants. A concluding inquiry could monitor the sustainability of these efforts in the long view.
